# How can we reduce the ischemic time for forearm replantation? Tips to simplify the bone fixation

**DOI:** 10.1186/s12891-023-06862-4

**Published:** 2023-09-18

**Authors:** Dong Hee Kim, Hyo Seok Jang, Sang Ho Kwak, Sung Yoon Jung, Jongmin Jeon, Hak Sang Kim, Sang Hyun Lee

**Affiliations:** 1 Departments of Orthopaedic Surgery, Samsung Changwon Hospital, Sungkyunkwan University School of Medicine, Changwon, Republic of Korea; 2 Department of Orthopaedic Surgery, Inje University Haeundae Paik Hospital, Inje University College of Medicine, Busan, Republic of Korea; 3 Department of Orthopaedic Surgery, SNU Seoul Hospital, Seoul, Republic of Korea; 4 Department of Orthopaedic Surgery, College of Medicine, Dong-A University Hospital, Busan, Republic of Korea; 5 Department of Orthopaedic Surgery, Medical Research Institute, Pusan National University Hospital, Pusan National University School of Medicine, Busan, Republic of Korea

**Keywords:** Ischemic time, Bone fixation, Amputation, Replantation, Forearm

## Abstract

**Purpose:**

Ischemic time is a key factor in satisfactory functional results after forearm replantation. In this study, we provide a detailed description of our surgical technique, the temporary screw plate fixation technique, which aims to reduce ischemic time.

**Methods:**

From June 2007 to June 2017, we performed a retrospective study of 20 patients who underwent forearm replantation. Eighteen cases involved male patients, and their mean age was 46 years. The mechanism of injury was roller injuries in 5 cases, power saw injuries in 3 cases, traffic accident in 7 cases, rope injuries in 2 cases, machinery injuries in 2 cases, and crushing injuries by rebar beam in 1 case.

**Results:**

A total of 20 replantation patients survived. According to injury level, there were 3 cases of the proximal type, 11 cases of the middle type, and 6 cases of the distal type. The average time to revascularization was 331 min. The total operation time was, on average, 5.73 h. In the rest of the 18 cases, the temporary screw plate fixation technique was performed, and the average time required for bone shortening and plate fixation was 38.3 min.

**Conclusions:**

To reduce ischemic time, we need a plan that progressively reduces time at each stage. Among our tips, temporary screw plate fixation can reduce the initial bone surgical operation to < 40 min, does not have many complications, and can be used as definitive surgery. This method for bone fixation should be considered as a strategy to actively reduce operation time during forearm replantation.

**Level of evidence:**

Retrospective study, Level III.

## Introduction

In the past, a survival rate of 74%–100% was reported in patients with upper limb replantation. However, recent studies published after the year 2000 reported survival rates of 94%–100% for major upper extremity replantation [1–7]. The functional results have also improved over time, with post-operative functional level now exceeding good/excellent in 50% of cases (Table 1) [1–4, 6, 7]. Considering that most cases involve younger, active patients, and there are lifetime financial benefits that could be achieved by limb salvage [6, 8, 9]. However, replantation requires hospital resources, high medical costs, and a high level of microsurgical technique. Considering the complications and costs, the decision between major limb replantation and revision amputation is difficult for both surgeons and patients [10].

**Table 1:** Case series for forearm replantation

**Authors**	Year	Case	Injury level	Mean age(Yrs)	Ischemic time (mean, Hrs)	Bone fixation technique	Bone shortening(Cm)	Survival(%)	Outcomes	Follow-up period
**Atzei A et al**	2005	10	Forearm	40.1	5.4	K-wire:4	ㅡ	100	Chen`s Grade II: 1	56.4 months
						Plate:3			Chen`s Grade III: 4	
						Ext fixtor:3			Chen`s Grade IV: 5	
**Sabapathy SR et al**	2007	20	Wrist:4	27	6	Plate:16	ㅡ	100	Chen`s Grade I: 3	37 months
			Forearm:12			K-wire:4			Chen`s Grade II: 9	
			Elbow & Arm: 4						Chen`s Grade III: 6	
									Chen`s Grade IV: 2	
**The Hoang N et al**	2009	10	Forearm	21.2	9.8	Plate:3	2.5 ~ 4	100	Chen`s Grade I: 1	12 ~ 42 months
						K-wire& circulage wiring: 7			Chen`s Grade II: 3	
									Chen`s Grade III: 3	
									Chen`s Grade IV: 3	
**Sugan TS el al**	2009	26	Transmatacarpal:6	26	6.5	Plate:13	3.72(5.22)	100	Chen`s Grade I: 8	11.3 yrs
			Wrist:4			K-wire:12			Chen`s Grade II: 9	
			Firearm:5			Ext fixtor:1			Chen`s Grade III: 3	
			Elbow: 4						Chen`s Grade IV: 6	
			Arm:7							
**Cavadas PC el at**	2009	27	Wrist:10	35.3	Primary: 4.25	Plate:17	ㅡ	100	Chen`s Grade I: 6	ㅡ
			Forearm:7		^a^Secondary: 3.42	K-wire: 10			Chen`s Grade II: 9	
			Elbow:1						Chen`s Grade III: 13	
			Arm:10						Chen`s Grade IV: 0	
**Leclere FM et al**	2012	11	Forearm: 9	43.4	5.85	Plate: 10	4.72	91	Chen`s Grade I: 4	7.5 yrs
			Elbow: 2						Chen`s Grade II: 3	
									Chen`s Grade III: 2	
									Chen`s Grade IV: 1	
									Amputation: 1	
**Gulgonen A. & Ozer K**	2011	9	Forearm: 7	24	3.5	ㅡ	ㅡ	100	Chen`s Grade I: 5	18 yrs
			Elbow: 2						Chen`s Grade II: 2	
									Chen`s Grade III: 2	
									Chen`s Grade IV: 0	
**Laing TA et al**	2012	19	Arm: 6	30	4.24	Plate:11	ㅡ	90	Tamai Score Good: 7	ㅡ
			Elbow:1			K-wire:3			Tamai Score Fair: 7	
			Forearm:7			Longitudinal Steinmann pins:5			Tamai Score Poor:3	
			Wrist:6						Exam impossible:1	
									Failed:2	

^a^Time between temporaray cather cessation to artery anastomisis

Previously published articles have investigated the most important factors affecting satisfactory functional outcomes in the replantation of the upper extremities. Patient factors including age, smoking, and anticoagulation have been discussed in terms of replantation outcomes, as have injury factors like the mechanism or level of injury [1, 6, 11]. In particular, ischemic time is the most often mentioned factor in the results of forearm replantation, and obtaining revascularization within 6 h of the injury generally results in good outcomes [1]. In order to reduce ischemic time during surgery, it is especially important to reduce the time required for debridement, exploration, and bone fixation, which are the steps before artery anastomosis.

There is controversy about the appropriate surgical sequence and method for major limb replantation. However, in a situation where ischemic time must be reduced, surgery is performed based on each surgeon's experience and usual skills of the surgeon that only they have. We suggest several tips for reducing ischemic time based on a conventional surgical technique in this paper and report our results.

## Patients and methods

This study was approved by the institutional review board of the Medical Research Institute at Pusan National University. All procedures were performed in accordance with the relevant guidelines and regulations. From June 2007 to June 2017, we performed a retrospective study of 20 patients who underwent forearm replantation. The exclusion criterion was multilevel forearm amputation. The surgeries were performed solely by one surgeon. Eighteen cases involved male patients, and their mean age was 46 years. Twelve patients were smokers and 2 patients were heavy drinkers. The detailed demographic characteristics of the patients are summarized in Table 2.

**Table 2:** Our series of 20 major upper-arm replantation Summary of Patient`s demography, surgical process and surgical outcomes

**Sex/Age**	**Level of Amputation**	**Injury Mechanism**	**Injury pattern**	**Time from injury to ER (min)**	**Time from begining to artery anastomosis(min)**	**Time to revascularixation**	**Time for bone shortening and plate fixaton**	**Total operation time(Hr)**	**Bone fixation: First stage**	**Bone fixation: Second stage**	**Secondary procedure**	**Chen’s grade**
M/42	Distal	Machine damage	Sharp cut	60	95	305	30	6	4-hole plate + EF	6-hole plate	-	Excellent
M/60	Proximal	Traffic Accident	Crushed	120	60	370	30	4.5	5-hole plate + EF	-	Skin graft	Fair
M/28	Distal	Power Saw	Sharp cut	60	85	355	30	5	4 hole plate + EF	6-hole plate	-	Good
M/43	Middle	Traffic Accident	Avulsion	60	60	360	60	5	6-hole plate + EF	-	ALT flap, Sural nerve graft(Median nerve)	Poor
M/47	Proximal(Elbow Joint)	Traffic Accident	Crushed	90	125	305	ㅡ	6	EF	-	Skin graft	Poor
M/49	Middle	Roller injury	Crushed	90	85	295	30	4.5	4-hole plate + EF	6-hole plate	ALT flap, Sural nerve graft(median nerve)	Poor
M/56	Distal	Power Saw	Sharp cut	60	85	295	30	6.5	4-hole plate + EF	-	Median nerve neurolysis	Good
M/53	Distal	Traffic Accident	Crushed	120	125	425	60	6.5	4-hole plate + EF	6-hole plate	ALT flap	Fair
M/25	Middle	Traffic Accident	Crushed	90	90	360	30	7.5	5-hole plate + EF	-	Skin graft	Excellent
M/68	Middle	By rebar beam	Crushed	30	95	375	30	6.5	4-hole plate + EF	6-hole plate	Skin graft	Fair
M/45	Middle	Roller injury	Avulsion	60	55	335	60	7.5	4-hole plate + EF	6-hole plate	Skin graft	Fair
M/46	Middle	Roller injury	Crushed	60	95	365	30	7.5	4-hole plate + EF	6-hole plate	Skin graft	Fair
M/56	Distal	Machine damage	Avulsion	150	145	355	30	7	4-hole plate + EF	-	-	Fair
M/60	Proximal(Elbow Joint)	Rope injury	Avulsion	60	60	240	ㅡ	6	Steinmann pin,EF	-	Dital bicep reconstruction, Vascularized ulnar nerve graft(ulnar nerve)	Good
M/23	Middle	Traffic Accident	Avulsion	60	90	300	30	6	5-hole plate + EF(Distal ulnar resection)	distal radius volar plate	Skin graft	Fair
M/67	Middle	Roller injury	Crushed	60	90	300	60	4.5	4-hole plate + EF	-	Skin graft	Poor
F/46	Middle	Traffic Accident	Avulsion	60	95	455	60	4.5	4-hole plate + EF	6-hole plate	ALT flap, Skin graft	Fair
F/62	Middle	Power Saw	Crushed	120	55	295	30	3.5	4-hole plate + EF	6-hole plate	Skin graft	Good
M/48	Middle	Rope injury	Crushed	120	90	300	30	5	4-hole plate + EF	6-hole plate	Skin graft	Fair
M/46	Distal	Roller injury	Avulsion	60	60	245	30	5	4-hole plate + EF	6-hole plate	-	Good

*EF* External fixator, *ALT flap* Anterolateral thight flap, *O* Performed

There were 3 cases of proximal-type injury, 11 cases of the middle type, and 6 cases of the distal type. The mechanism of injury was crushing in 10 cases, avulsion in 7 cases, and guillotine amputation in 3 cases. One patient underwent arm replantation surgery with amputation of the right lower leg. First, we performed arterial connection after bone fixation of the arm, followed by a bone fixation on the leg side, and then connected the artery. Then, the arm vein and the leg vein were connected. One patient had an epidural haemorrhage but did not require neurosurgery. Three patients had fractures of the ipsilateral humerus (shaft fracture in 2 patients, distal humerus fracture in 1 patient). One patient had a forearm fracture on the opposite side. We performed open reduction and internal fixation 2 weeks after replantation.

All patients were evaluated retrospectively. We investigated the time from injury occurrence to arterial anastomosis. We also investigated the time required for bone shortening and fixation during surgery.

### Surgical technique of the replantation

#### Wound debridement and exploration

**Fig. 1:**
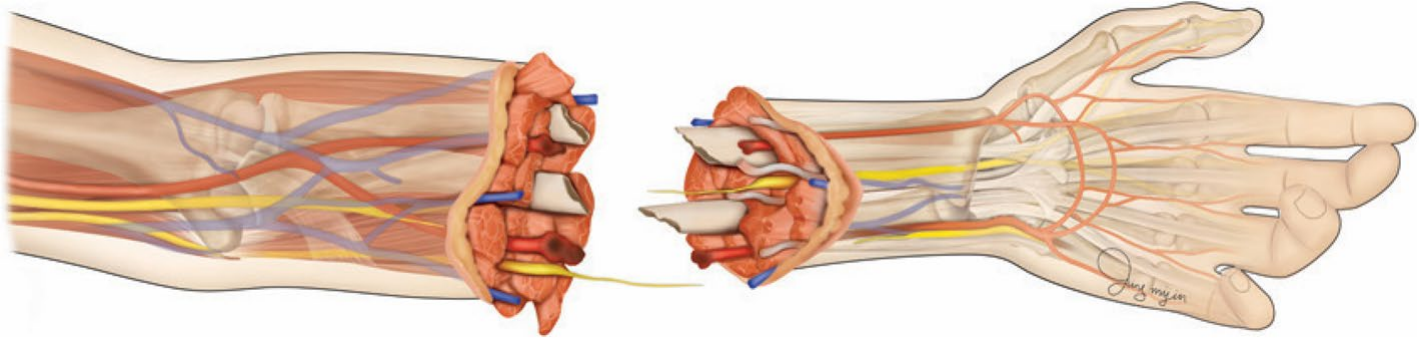
Forearm amputation

Since most included patients underwent amputation due to severe trauma and there were many foreign materials in the wound that could cause infection, the damaged area was debrided while removing foreign substances (Fig. 1). In general, each important structure was systematically tagged for the surgeon’s convenience for subsequent surgery, but we first searched for arteries to reduce ischemic time. The arteries proximal to the amputation site show pulsating arterial flow and can easily be found, while the arteries of amputated extremities are identified based on anatomical location and marked with 6–0 nylon thread. Veins and nerves were not identified so as to reduce ischemic time.

#### Bone shortening and fixation: minimal dissection and small plate

**Fig. 2:**
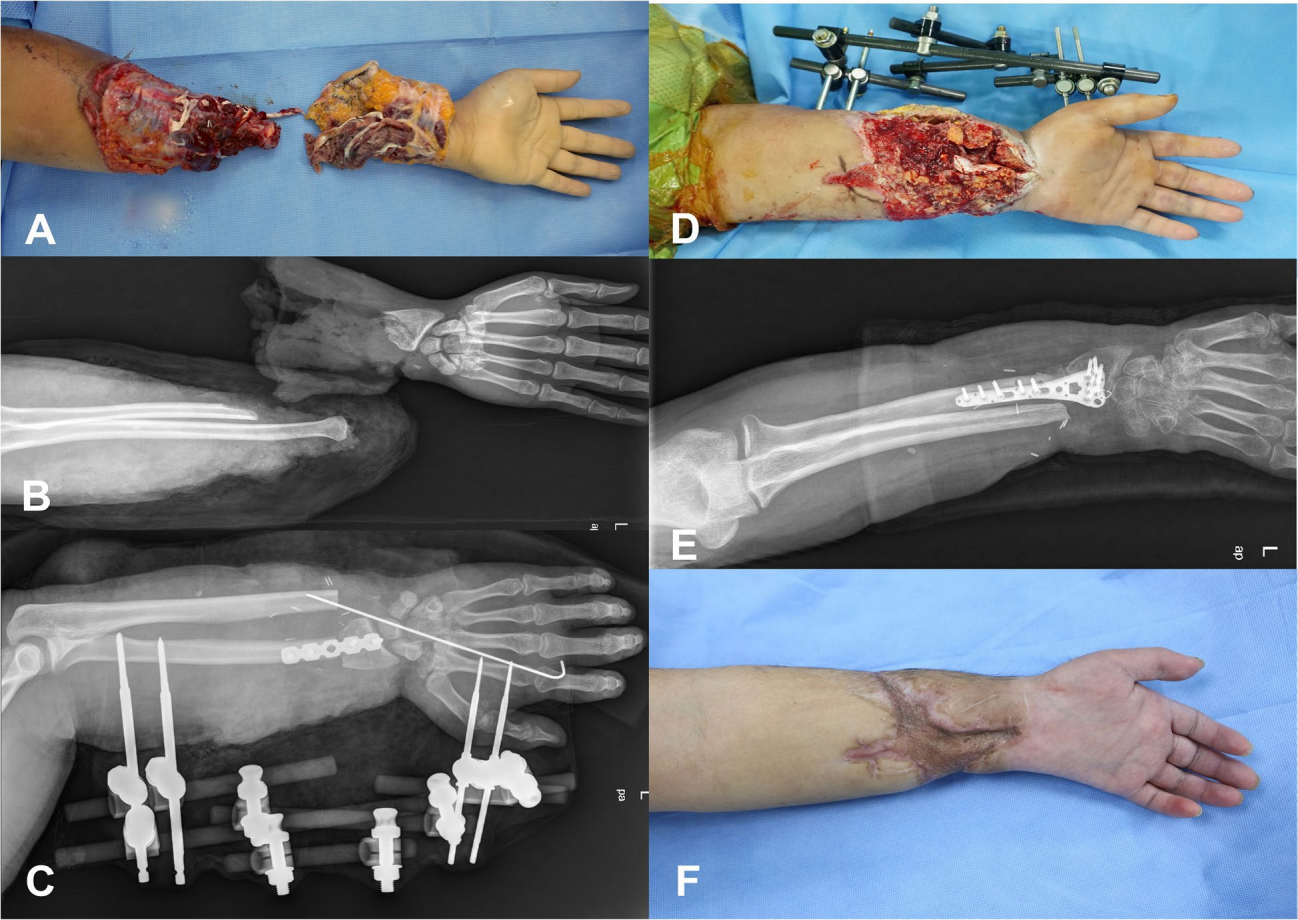
**A** A 23-year-old male patient experienced traumatic amputation of the middle of the forearm of the left arm due to a traffic accident. **B** The X-ray shows that the injury occurred at the distal radius and the ulnar bone is disarticulated. **C** We performed bone fixation to the distal radius with a 5-hole plate and performed resection of the distal ulna, followed by an external fixator. **D** Due to severe skin loss at the amputation site, a skin graft is planned for the subsequent surgery. **E** During the second surgery, we replaced the plate with a volar locking plate at the distal radius. **F** This photo was taken 3 months after the initial trauma, and subsequent rehabilitation therapy was performed

The two-stage bone-fixation technique used by the authors involves placing a 4- or 6-hole plate for bone fixation, performing vascular anastomosis, and then using an external fixator to complete bone fixation (Fig. 2). We determined the length of the metal plate based on the length of exposed bone after bone shortening. We cut the comminuted or oblique fractures at the amputated site vertically using a saw and fixed the plate, thereby reducing the time to dissected the fractured area. A 4- to 6-hole recon-plate was mainly used, and only a single radius or ulna was fixed; then, blood circulation was restored by connecting the arteries. In some cases, an external fixator was additionally performed if the plate alone was insufficient to fix fracture site at the end of all surgical procedures.

#### Vessel, nerve, muscle, and tendon repairs

Typically, direct anastomosis and vein graft anastomosis are used to connect arteries, but we preferred direct anastomosis, which requires less operation time. If radial and ulnar arteries are severely damaged, shortening of the bone can be minimized by crossing and connecting these two arteries. Through appropriate bone shortening, we tried to suture the 3 major nerves with direct suture techniques without a nerve graft. Most of the nerves in the amputated area were stretched, which made it difficult to suture them; therefore, only healthy nerves were connected for sensory recovery and marked for nerve transplantation later. On myography, muscle at the amputation site with a high possibility of infection and necrosis were removed as much as possible, and the site’s connecting vessels covered using the proximal muscle to prevent damage. Also, proximal muscles in good condition were selected and connected to the tendons of the amputated part. At this time, the second, third, fourth, and fifth finger flexor tendons were collected and connected, and the tendon of the thumb was connected separately. Extensors were also collected and connected to muscles in good condition. At the amputation site, the skin was first covered to protect the blood vessel connections, and the skin was sutured so that the bones or the fixed metal plate were not exposed.

#### Soft tissue coverage and second-stage bone fixation

Most wounds caused by acute bone shortening in the early stage could be covered with sufficient soft tissue. For additional soft tissue defects, a secondary bone fixation surgery was performed 4–6 weeks after surgery. In our patients, most underwent skin grafts, free flaps were used in some during the second surgery. For soft tissue damage, a skin graft was performed in 10 cases, ALT was done in 3 cases, and both ALT and skin graft were done in 1 case.

The second stage of bone fixation was performed 4–6 weeks after surgery if the amputated area had survived and the wound was covered to some extent. For some patients who initially received a 3.5-mm diameter 4-hole recon plate, a 6-hole plate was subsequently fixed. In cases where an external fixator was placed, the external fixator was removed and a 6-hole plate was placed. Secondary bone fixation could not be performed in some cases due to infection or the general condition of the patients. If there was a gap at the fracture site, iliac bone grafting was performed.

Nerve grafts mainly used sural nerves and were performed 3–6 months after surgery. Neurolysis was performed in 1 case, and a sural nerve graft was performed in 2 cases where the median nerve could not be connected. In 1 case, a vascularized ulnar nerve graft was placed at the ulnar nerve.

Total active motion of the wrist was measured at the final follow-up visit. We evaluated touch sensation using a pinprick test; it was also classified by Chen's grade in terms of strength and sensation.

## Results

A total of 20 patients were included in final follow-up. The mean follow-up period was 27.9 (range, 10–81) months. The average time to revascularization was 331 min (range, 240–425 min). The average interval between amputation and patient arrival time to the emergency department was 79.5 min (range, 30–120 min), and the average time from checking the general condition in the emergency room to entering the operating room was 89 min (60–120 min). The mean anaesthesia time was 32 min. It took an average of 277 min from the patient’s injury occurrence to preparation for surgery. The total operation time was, on average, 5.73 h (range, 4.5–7.5 h), and the time necessary to finish arterial anastomosis was 87 min (range, 60–145 min). Among proximal-level injuries, 2 cases of amputation near the elbow joint were fixed using an external fixator and rush pin. In the remaining 18 cases, plating and external fixation were performed, and the average time required for bone shortening and plate fixation was 38.3 min (range, 30–60 min). The mean amount of bone shortening achieved was 2.97 cm (range, 0–7 cm), and external fixation was performed in all patients. Plate conversion or additional plate placement occurred in 12 of 18 patients initially underwent plating and external fixation.

All 20 cases survived to final follow-up time and achieved bone union. The mean rate of total active motion at the wrist was 54% relative to the unaffected side (range, 20%–85%). In 11 cases, touch sensation was recovered by pinprick test at the median or ulnar territory. Classification by Chen's grade showed 3 cases of type I, 4 cases of type II, 3 cases of type III, and 4 cases of type IV recovery (Table 2).

## Discussion

**Fig. 3:**
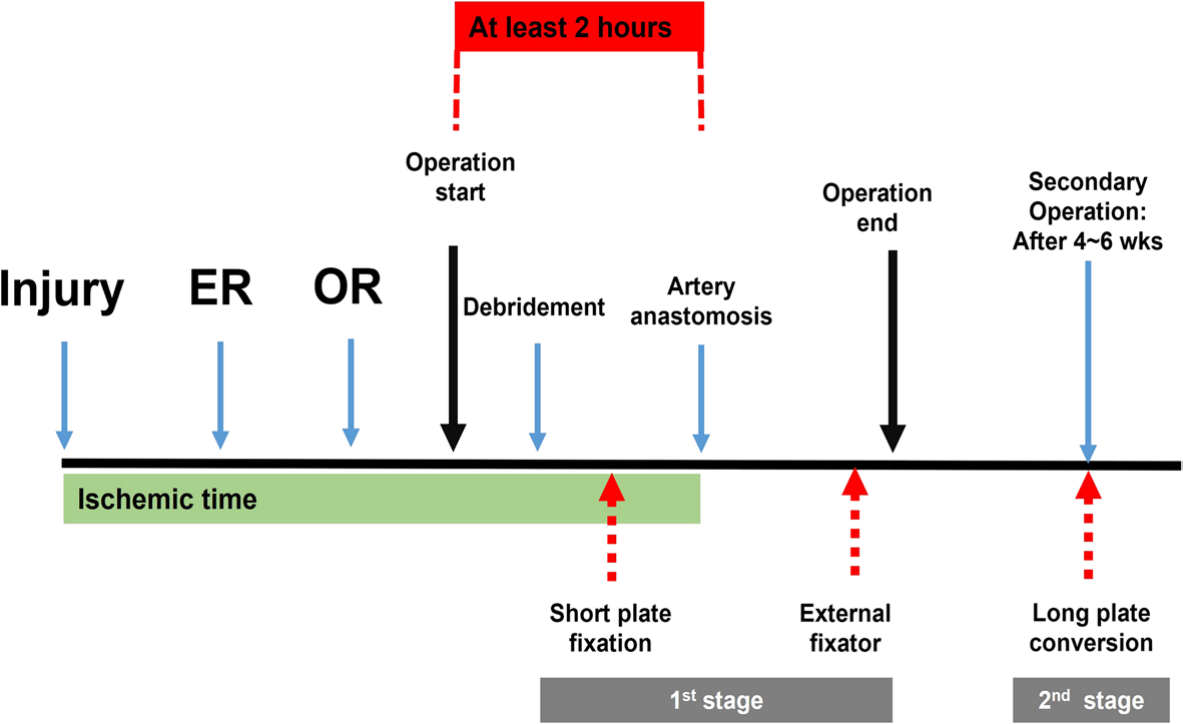
Process of forearm replantation

This study focused on reducing ischemic time from operation onset to arterial anastomosis in forearm replantation surgery. Prolonged ischemic time can lead to irreversible damage to the skeletal muscle, making revision and replantation impossible and adversely affecting the outcomes of surgery [1, 12]. In this study, we reduced the ischemic time during surgery to less than 2 h by simplifying bone fixation. In clinical practice, if we consider the various processes from amputation to the start of surgery, there is not enough time left for surgeon (Fig. 3) [13]. According to our data, the average interval between amputation and patient arrival to the emergency room was 89.5 min (range, 30–120 min), and an average of 89 min (range, 60–120 min) from check in to preparation for surgery. It took an average of 32 min to anesthetize; therefore, we found an average of 2 h from the begin of the operation as a gold time. Therefore, surgical debridement including bone fixation and arterial anastomosis should be performed within 2 h. We focused on bone shortening and minimal bone-fixation techniques during the 2-h golden time. Our two-stage bone-fixation technique allowed primary bone fixation to be done on average < 40 min from the start of surgery, thereby reducing the total ischemic time to < 6 h.

Major limb replantation outcomes are significantly affected by ischemic time, as well as by injury level. The injury level in forearm amputation determines how much muscle mass is included in the amputated limb and affects the outcome due to reperfusion injury after surgery [1, 2, 14, 15]. Therefore, there is also an argument that the ischemic time should be considered accordingly [1]. In general limb amputation, blood circulation must be restored within 6 h to obtain good results, or within 4 h in proximal amputation [1]. Most of the research reporting functional outcomes also reports better results for mid-forearm- and distal-level amputation than for proximal amputation, which is considered to be in line with this [14, 16]. Therefore, a long warm ischemic time prior to surgery and proximal forearm amputation are indications for the use of our technique.

There are other methods to increase the success rate of surgery and save time during surgery, in addition to reducing ischemic time. For example, connecting the stump to an extra-anatomical-area artery or creating and maintaining cold ischemia with catheters that use cold saline or blood maintain blood circulation [7, 17–19]. Connecting the limb to a different artery to maintain blood circulation is an effective way to buy time, but it increases surgery time by adding another procedure. Injecting cold saline into the stump using a catheter can help lower core temperature and reduce complications. However, as Sabapathy [20] pointed out, using cold saline is not a decisive way to reduce ischemic time. We think that these methods are more appropriate in cases of extensive contamination or multiple amputations where debridement takes longer or limb amputation occurs in multiple areas. It is generally difficult to apply these methods to typical traumatic amputations. We believe that reducing the time for bone fixation is a more efficient approach to surgical procedures that require joint connection.

Bone fixation for forearm transplantation should reduce ischemic time while providing minimal fixation force. There are only a few studies describing the fixation method in detail among available research on major limb replantation [1–4, 6, 7, 14, 16, 21, 22]. In general, K-wire or plate or external fixation is used for bone fixation [1–4, 6, 7]. However, in the case of forearm amputation, K-wire fixation is not applicable and even unstable in the radius and ulnar due to small diameters. Also, fixation of both bones using only an external fixator is very inconvenient for dressing and it is difficult to maintain the external fixator until achieving bone union. Therefore, we used a rapid bone fixation technique using a 4 or 6-hole recon plate, which is more stable than using a K-wire and is sufficient for short-term fixation, with almost no additional periosteal strip required, enabling preservation of the periosteal circulation. This technique is advantageous in patients who have already had a long warm ischemic time and those with proximal forearm amputation.

**Fig. 4:**
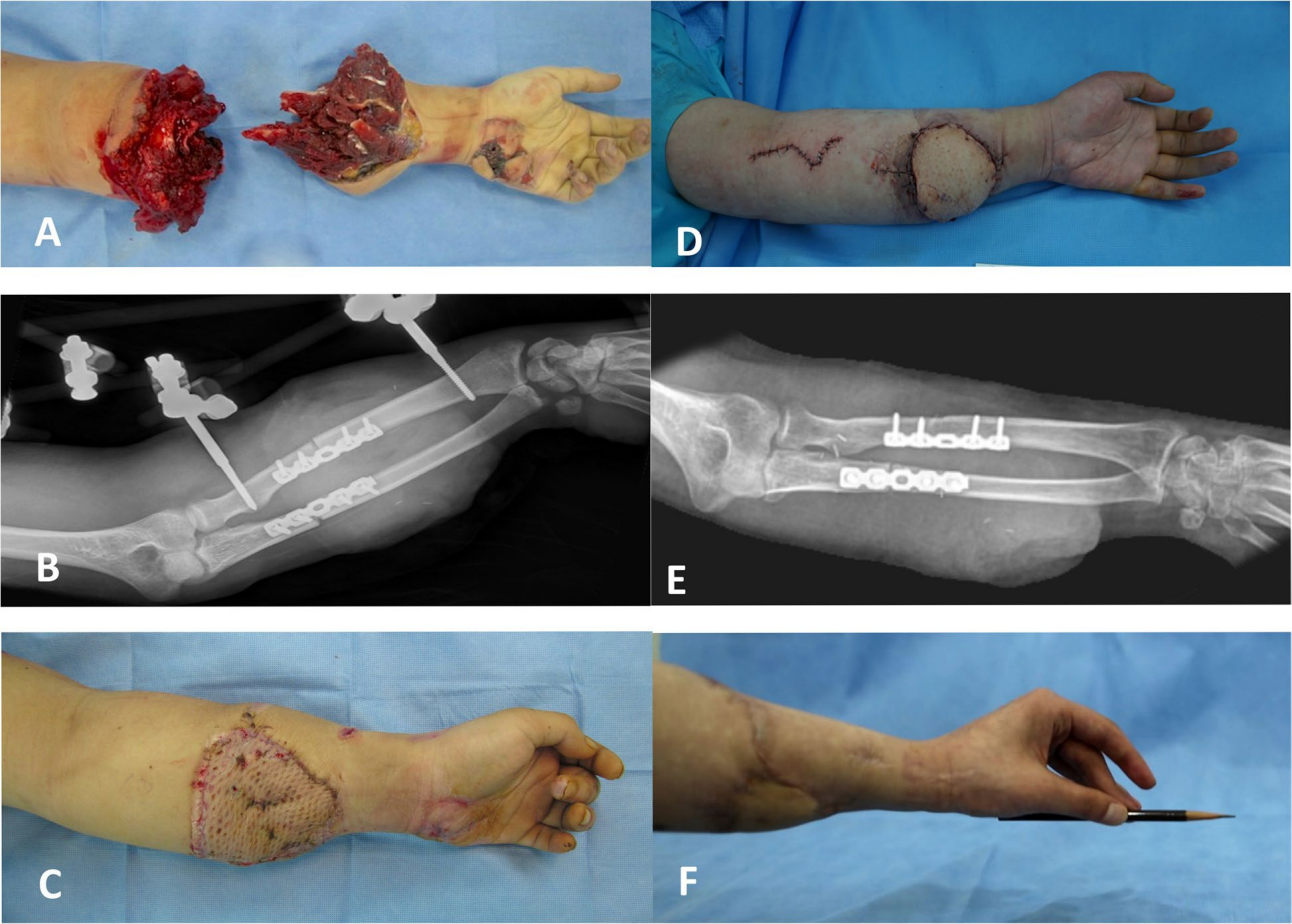
**A** Photographic showing a 24-year-old male amputated forearm after a car accident. **B** We used a 5-hole recon-plate and external fixator for bone fixation during replantation surgery.It took 120 min from the start of the operation to artery anastomosis, and the total ischemia time was 7 h. The time required for bone shortening and plate fixation was 30 min. **C** The external fixator was removed 6 weeks after surgery and a skin graft was done. **D** For hand function, a free functional muscle graft using the gracilis muscle was performed 15 months after surgery. **E** Bone union was achieved without secondary bone fixation. A free functional muscle graft using the gracilis muscle was performed 15 months after surgery. **F** At the last follow-up at 19 months, Chen's grade II excellence (total range of motion ≥ 60%, muscle str ≥ M4, sensibility ≥ 2, back to work) was found.ength

Temporary screw plate fixation can be applied to most cases as a form of damage control. It allows for primary bone fixation to be achieved in a short amount of time, reducing the ischemic time during surgery. However, it should be noted that the total surgery time may not necessarily decrease. Instead, this technique focuses on reducing the time to arterial anastomosis. The problem with temporary screw plate fixation is that it requires an additional surgery. In most patients, when the replanted limb is stabilized and the patient's general condition improves, additional surgery for soft tissue is required [23]. Then, secondary bone fixation can be performed as well. In some patients, the second stage could not be completed due to comorbidities or infection at the surgical site, but sufficient bone union could still be achieved in these patients (Fig. 4). Accordingly, temporary screw plate fixation technique can be applied to most cases as a form of damage control, and, in some cases, it was substituted for definitive surgery without additional reconstruction.

The main limitations of this study were that it was retrospective and involved a small number of cases. As a result, we were unable to form a control group or detailed operation times. Additionally, there was variation in the type of hardware used, which may have affected the results of the study. Furthermore, while temporary screw plate fixation can be useful in reducing ischemic time, its potential complications require further investigation. Future studies with a larger sample size and a control group are needed to establish the safety and effectiveness of this technique.

## Conclusion

In conclusion, temporary screw plate fixation reduces the time to arterial anastomosis by simplifying bone fixation. Our technique could not reduce the total operation time; however, it can be considered an option for reducing the initial operation time in select patients requiring major forearm replantation.

## Data Availability

The datasets used and analysed during the current study are not publicly available due to lack of participant consent to share their data but are available from the corresponding author upon reasonable request after ethical considerations are met.
